# Supplementary material for: Exploring cross-tissue DNA methylation patterns: blood–brain CpGs as potential neurodegenerative disease biomarkers
Source: Commun Biol. 2024 Jul 26;7:904. doi: 10.1038/s42003-024-06591-x (PMC11282059; doi:10.1038/s42003-024-06591-x)
Supplement: Supplementary file 2 — Supplementary Information [file 42003_2024_6591_MOESM2_ESM.pdf]

Supplementary information

Supplementary Table 1 - Description of the data sets from the GEO database and used in the present work

				Cases			Controls			
**GEO ID**	**Cohort**	**Disease**	**Sample Type**	**N**	**Age (years)**	**Sex (female/male)**	**N**	**Age (years)**	**Sex (female/male)**	**Reference**
GSE153712	Australian Imaging, Biomarkers, and Lifestyle (AIBL)	Alzheimer	blood	161	>60	91/70	471		272/199	NABAIS et al., 2021[1]
GSE72778	University of California, Los Angeles (UCLA) Brain Research Institute	Alzheimer	brain	18[a]	58-114	13/5	21[b]	15-93	6/15	HORVATH et al., 2016[2]
GSE111629	University of Auckland Parkinson's Environment and Gene	Parkinson	blood	335	36 - 90	138/197	237	35-93	111/126	CHUANG et al., 2017[3]
GSE195834		Parkinson	brain	40	70-87	19/21	38	59-87	18/20	VISHWESWARAIAH et al., 2022[4]
GSE41273		Fragile X Syndrome	blood	9	01-48	-/9	53	03-18	-/53	ALISCH et al., 2013[5]
GSE106648		Multiple Sclerosis	blood	140	16-66	98/42	139	20-65	104/35	KULAR et al., 2018[6]

[a] Resulting in n= 125 samples (1-12 samples/patient)
[b] Resulting in n=135 samples (3-8 samples/patient)

Supplementary information

Supplementary Table 2: Information about the nine hypermethylated CpGs in blood DNA from Alzheimer patients in comparison to healthy controls

CpG	Chr	Gene	CpG_features	padj	Change	deltaBeta (mean)
cg06335143	1	*ZYG11A*	Body-island	0.00483	Increased	0.02024
cg06639320	2	*FHL2*	TSS200-island	0.04656	Increased	0.02027
cg07553761	3	*TRIM59*	TSS1500-island	0.00333	Increased	0.03184
cg06493994	6	*SCGN*	1stExon-island	0.00002	Increased	0.01559
cg21572722	6	*ELOVL2*	TSS1500-island	0.00072	Increased	0.01667
cg16867657	6	*ELOVL2*	TSS1500-island	0.00143	Increased	0.02413
cg24724428	6	*ELOVL2*	TSS1500-island	0.00229	Increased	0.02455
cg19904058	10	*LOC619207*	Body-island	0.04629	Increased	0.03305
cg03032497	14		IGR-island	0.03888	Increased	0.02048

Chr: chromosome; CpG_features: CpG position according to “gene-CpG island”; 1stExon: CpG located in the first exon of a gene; TSS1500: 1500 nucleotides upstream of transcription start site; body: gene body; IGR: intragenic region; TSS200: 200 nucleotides upstream of transcription start site; padj: Benjamini-Hochberg adjusted p-value.

ISSN 2399-3642 (online)

Supplementary information

Supplementary Table 3: Information about the nine hypomethylated CpGs in blood DNA from Parkinson patients in comparison with healthy controls

CpG	Chr	Gene	CpG_features	padj	Change	deltaBeta (mean)
cg08347373	2	*CD302*	Body-shore	0.03992	Decreased	-0.03142
cg25821974	3	*RAB7A*	5'UTR-shelf	0.00412	Decreased	-0.03642
cg02368812	6	*NQO2*	3'UTR-opensea	0.00205	Decreased	-0.04215
cg11725581	7		IGR-shelf	0.00069	Decreased	-0.03996
cg17392018	7	*CALD1*	Body-opensea	0.03992	Decreased	-0.02757
cg14255824	9	*TJP2*	Body-opensea	0.03992	Decreased	-0.02652
cg21649013	12	*ULK1*	Body-shore	0.03992	Decreased	-0.01751
cg24904992	17	*TBC1D16*	5'UTR-shelf	0.04184	Decreased	-0.01578
cg12230709	19	*PRTN3*	TSS200-shelf	0.00205	Decreased	-0.02571

Chr: chromosome; CpG_features: CpG position according to “gene-CpG island”; body: gene body; 5´UTR located five prime untranslated regions; 3UTR: located three prime untranslated region; IGR: intragenic region; TSS200: 200 nucleotides upstream of transcription start site; padj: Benjamini-Hochberg adjusted p-value.

Supplementary information

Supplementary Table 4: Information about the differentially methylated CpGs in blood DNA from Multiple Sclerosis patients in comparison to healthy controls

CpG	Chr	Gene	CpG_features	padj	Change	deltaBeta (mean)
cg19864490	1	*FAM78B*	TSS1500-island	0,04544	Increased	0.0082
cg21245277	2	*ZNF804A*	1stExon-island	0,00990	Increased	0.0112
cg26328180	2		IGR-island	0,03713	Increased	0.0239
cg06027691	3	*CTDSPL*	Body-opensea	0,00474	Increased	0.0338
cg04521004	3	*SHOX2*	Body-island	0,01654	Increased	0.0171
cg03427905	3	*ADAMTS9*	Body-island	0,03290	Increased	0.0112
cg22488278	4	*ZFYVE28*	Body-opensea	0,00280	Decreased	-0.0282
cg09139047	6	*HLA-DRB1*	Body-island	0,00280	Increased	0.1619
cg11404906	6	*HLA-DRB1*	Body-shore	0,00280	Increased	0.1946
cg13910785	6	*HLA-DRB1*	Body-shelf	0,00280	Decreased	-0.1875
cg17369694	6	*HLA-DRB5*	3'UTR-opensea	0,00280	Decreased	-0.1579
cg22627029	6	*HLA-DRB6*	Body-opensea	0,00280	Decreased	-0.1928
cg16514085	6	*HLA-DRB1*	Body-island	0,00295	Increased	0.1783
cg18111114	6	*HLA-DRB5*	TSS1500-opensea	0,00295	Decreased	-0.0601
cg00886432	6		IGR-opensea	0,01545	Decreased	-0.0261
cg05383619	6	*HLA-DRB1*	Body-shore	0,02912	Decreased	-0.0755
cg15708909	6	*HLA-DRB5*	Body-shelf	0,03290	Increased	0.0703
cg10995422	6	*HLA-DRB6*	Body-opensea	0,03993	Decreased	-0.1646
cg00119778	6		IGR-opensea	0,04544	Decreased	-0.0151
cg18024368	6	*HLA-DQB1*	Body-island	0,04544	Increased	0.0260
cg24638099	6	*HLA-DRB6*	Body-opensea	0,04544	Decreased	-0.0951
cg04671932	7	*MAGI2*	TSS200-shore	0,03288	Increased	0.0098
cg11592503	7	*CNTNAP2*	TSS200-island	0,04107	Increased	0.0208
cg26181840	13	*RASA3*	Body-island	0,01329	Decreased	-0.0204
cg06452665	13	*TNFSF11*	1stExon-island	0,04590	Increased	0.0213
cg17594334	14	*PLEKHH1*	5'UTR-island	0,04480	Increased	0.0075
cg16927372	17	*ACCN1*	Body-shore	0,00474	Decreased	-0.0230
cg10137837	17	*BCL6B*	5'UTR-island	0,03288	Increased	0.0227

Chr: chromosome; CpG_features: CpG position according to “gene-CpG island”; TSS1500: 1500 nucleotides upstream of transcription start site; 1stExon: CpG located in the first exon of a gene; IGR: intragenic region; body: gene body; 3´UTR: located three prime untranslated regions; 5´UTR located three prime untranslated regions; TSS200: 200 nucleotides upstream of transcription start site; padj: Benjamini-Hochberg adjusted p-value.

ISSN 2399-3642 (online)

Supplementary information

Supplementary Table 5: Information about the differentially methylated CpGs in blood DNA from patients with X Fragile Syndrome in comparison to healthy controls.

CpG	Chr	Gene	CpG_features	padj	Change	deltaBeta (mean)
cg14602222	12	*RAD52*	Body-opensea	0.01410	Increased	0.1032
cg24678163	13	*THSD1P*	TSS1500-shore	0.02960	Decreased	-0.0645
cg15116481	19	*KLK15*	Body-island	0.01830	Decreased	-0.1067
cg02921434	X	*ASFMR1*	Body-island	0.00000	Increased	0.6979
cg04552106	X	*ASFMR1*	Body-island	0.00000	Increased	0.7272
cg04744025	X	*ASFMR1*	Body-island	0.00000	Increased	0.5470
cg05288927	X	*ASFMR1*	Body-island	0.00000	Increased	0.6189
cg07147350	X	*ASFMR1*	Body-shore	0.00000	Increased	0.4551
cg08434396	X	*ASFMR1*	Body-island	0.00000	Increased	0.7164
cg16783314	X	*ASFMR1*	Body-island	0.00000	Increased	0.6650
cg19741073	X	*ASFMR1*	Body-island	0.00000	Increased	0.7533
cg21274274	X	*ASFMR1*	Body-island	0.00000	Increased	0.6061
cg22417678	X	*ASFMR1*	TSS1500-opensea	0.00000	Decreased	-0.2076
cg22625568	X	*ASFMR1*	Body-shore	0.00000	Increased	0.6146
cg27064928	X	*ASFMR1*	Body-shelf	0.00000	Increased	0.3216
cg22675021	X	*CTAG2*	1stExon-opensea	0.00790	Decreased	-0.1156
cg02055404	X		IGR-island.	0.01710	Increased	0.0439
cg06307153	X	*ZNF75D*	5'UTR-island	0.04870	Increased	0.0695

Chr: chromosome; CpG_features: CpG position according to “gene-CpG island”; body: gene body; TSS1500: 1500 nucleotides upstream of transcription start site; 1stExon: CpG located in the first exon of a gene; IGR: intragenic region; 5′UTR located three prime untranslated regions; padj: Benjamini-Hochberg adjusted p-value.

Supplementary information

Supplementary Figure 1: Difference in average beta values between Parkinson's disease patients and healthy controls for each CpG, using DNA derived from blood

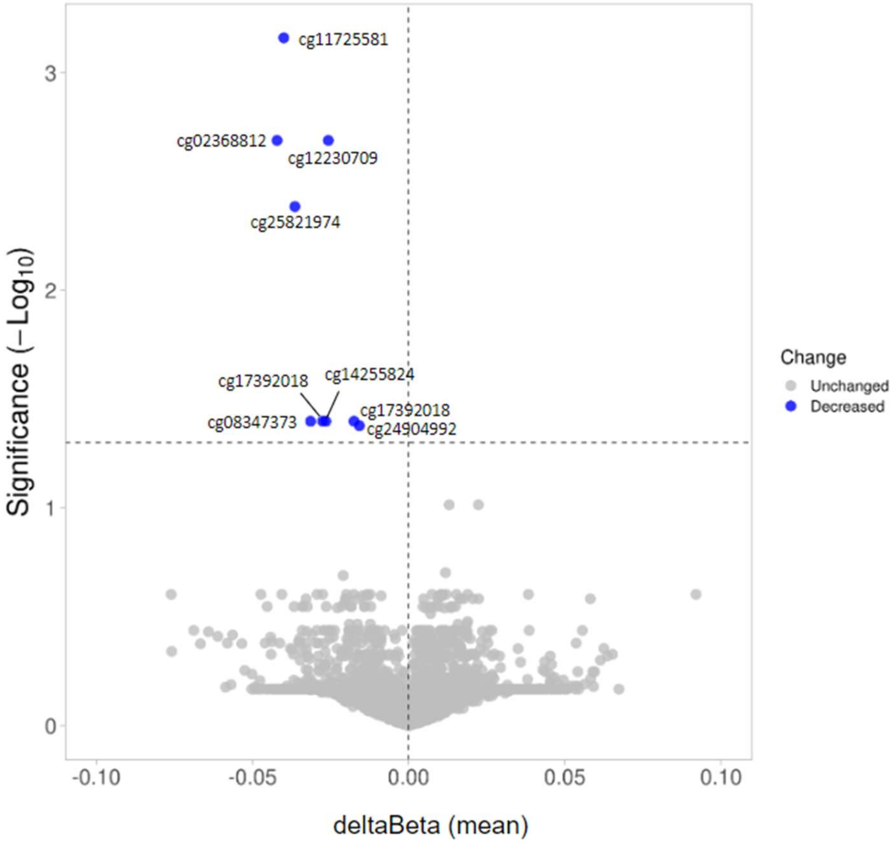

Supplementary Figure 1: Graph showing the distribution of average beta values for each CpG analyzed in DNA derived from blood samples of patients with Parkinson's disease in comparison to healthy controls; p-value after Benjamini-Hochberg correction. Were used n=335 biologically independent samples from patients and n=237 as controls.

ISSN 2399-3642 (online)

Supplementary information

Supplementary Figure 2: Difference in average beta values between Parkinson's disease patients and healthy controls for each CpG, using DNA derived from brain

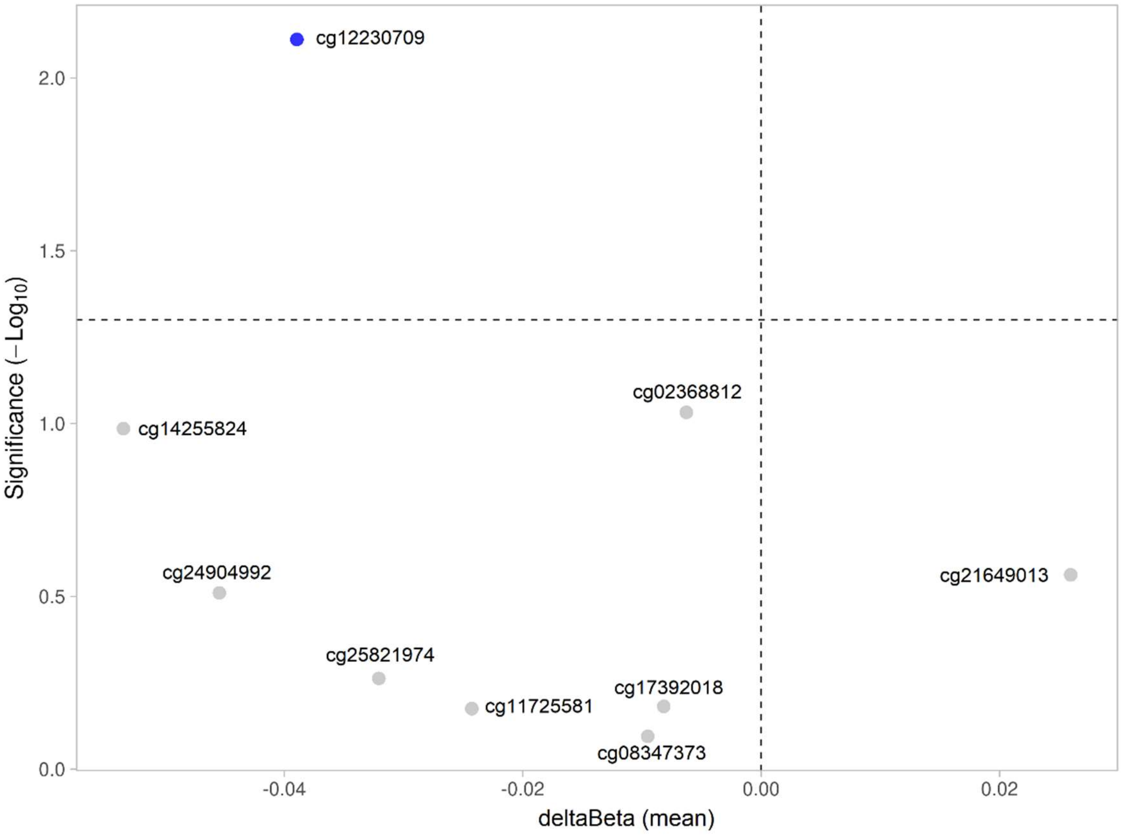

Supplementary Figure 2: Graph showing the distribution of average beta values for each CpG analyzed in DNA derived from brain samples of Parkinson's patients in comparison to healthy controls; p-value after Benjamini-Hochberg correction. Were used n=38 biologically independent samples from patients and n=40 as controls.

Supplementary information

Supplementary Figure 3: Difference in average beta values between Multiple Sclerosis patients and healthy controls for each CpG, using DNA derived from blood

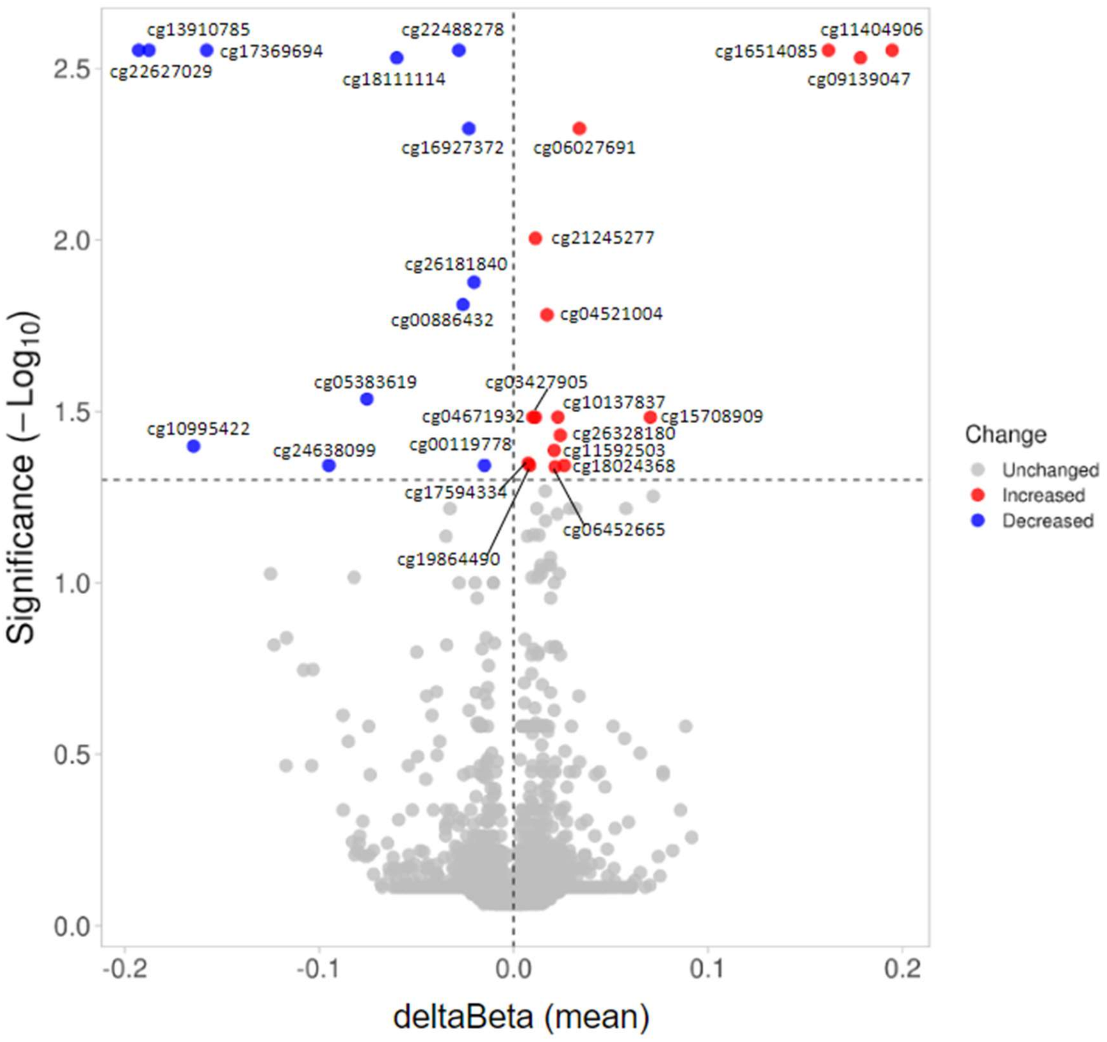

Supplementary Figure 3: Graph showing the distribution of average beta values for each CpG analyzed in DNA derived from blood samples of patients with Multiple Sclerosis in comparison to healthy controls; p-value after Benjamini-Hochberg correction. Were used n=140 biologically independent samples from patients and n=139 as controls.

ISSN 2399-3642 (online)

Supplementary information

Supplementary Figure 4: Difference in average beta values between Fragile X syndrome patients and healthy controls for each CpG, using DNA derived from blood

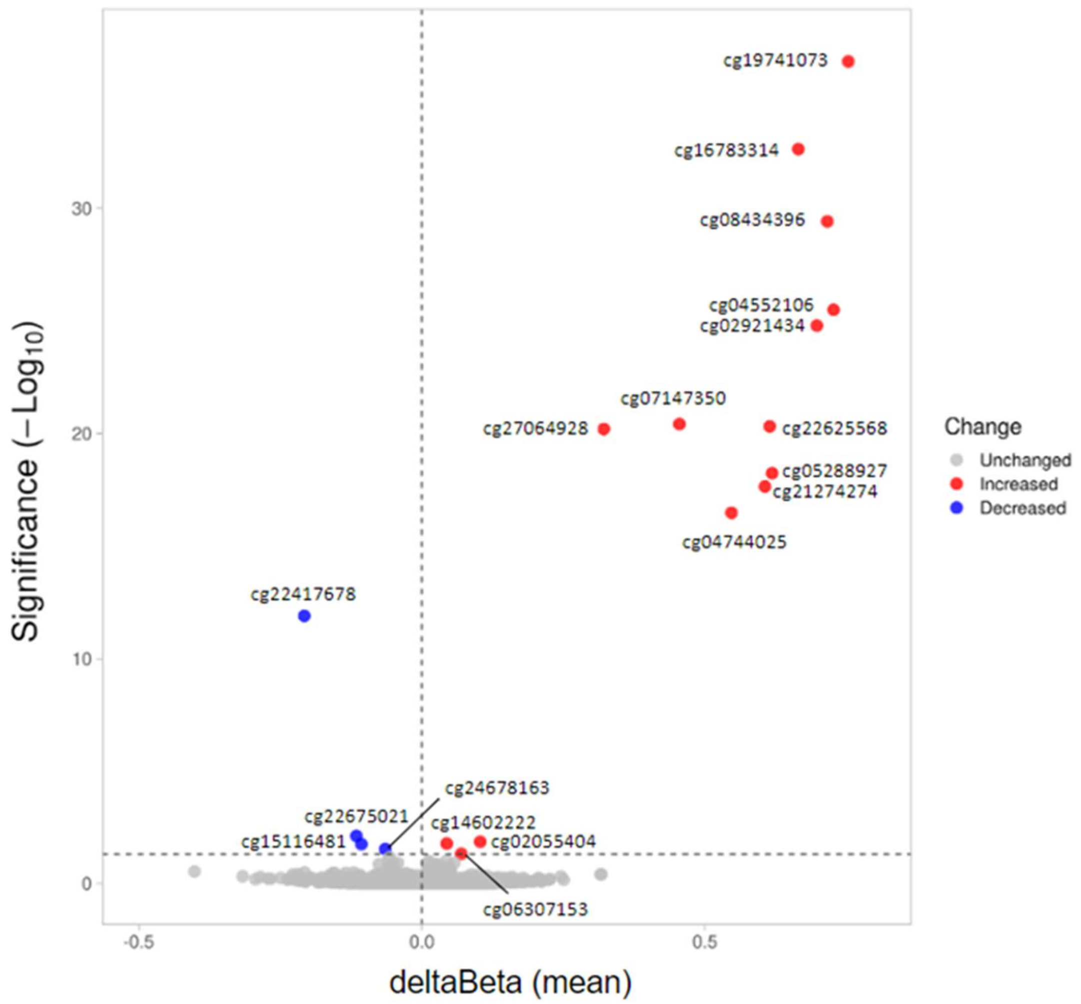

Supplementary Figure 4: Graph showing the distribution of average beta values for each CpG analyzed in DNA derived from blood samples of patients with Fragile X syndrome in comparison to healthy controls; p-value after Benjamini-Hochberg correction. Were used n=9 biologically independent samples from patients and n=53 as controls.

Supplementary information

Supplementary References

1. Nabais M. F. et al. Meta-analysis of genome-wide DNA methylation identifies shared associations across neurodegenerative disorders. Genome Biol. 22, 1, 90; 0.1186/s13059-021-02275-5. (2021)

2. Horvath, S. et al. Huntington's disease accelerates epigenetic aging of human brain and disrupts DNA methylation levels. Aging. 8, 7, 1485-1512. 10.18632/aging.101005 (2016).

3. Chuang Y. H. et al. Parkinson's disease is associated with DNA methylation levels in human blood and saliva. Genome Med. 9,1, 76; 10.1186/s13073-017-0466-5 (2017).

4. Vishweswaraiah S. et al. Methylated Cytochrome P450 and the Solute Carrier Family of Genes Correlate with Perturbations in Bile Acid Metabolism in Parkinson's Disease. Front Neurosci, 16,804261; 10.3389/fnins.2022.804261 (2022).

5. Alisch R.S et al. Genome-wide analysis validates aberrant methylation in fragile X syndrome is specific to the FMR1 locus. BMC Med Genet. 14, 18; 10.1186/1471-2350-14-18 (2013).

6. Kular L, Liu Y, Ruhrmann S, Zheleznyakova G et al. DNA methylation as a mediator of HLA-DRB1*15:01 and a protective variant in multiple sclerosis. Nat Commun. 9,1,2397; 10.1038/s41467-018-04732-5 (2018).
