# Supplementary material for: Exploring cross-tissue DNA methylation patterns: blood–brain CpGs as potential neurodegenerative disease biomarkers
Source: Commun Biol. 2024 Jul 26;7:904. doi: 10.1038/s42003-024-06591-x (PMC11282059; doi:10.1038/s42003-024-06591-x)
Supplement: Supplementary file 3 — Description of Additional Supplementary Files [file 42003_2024_6591_MOESM3_ESM.pdf]

Description of Additional Supplementary Files

File name: Supplementary Data 1

Description: information about 18,293 CpGs analyzed in this study and their correlation coefficient between blood and brain in each database.

File name: Supplementary Data 2

Description: The source data behind the volcano plots.
