# Supplementary material for: Exploring cross-tissue DNA methylation patterns: blood–brain CpGs as potential neurodegenerative disease biomarkers
Source: Commun Biol. 2024 Jul 26;7:904. doi: 10.1038/s42003-024-06591-x (PMC11282059; doi:10.1038/s42003-024-06591-x)
Supplement: Supplementary file 6 — Reporting summary [file 42003_2024_6591_MOESM6_ESM.pdf]

Corresponding author(s): MIGUEL ANGELO MARTINS MOREIRA

Last updated by author(s): Jul 3, 2024

Reporting Summary

Nature Portfolio wishes to improve the reproducibility of the work that we publish. This form provides structure for consistency and transparency in reporting. For further information on Nature Portfolio policies, see our Editorial Policies and the Editorial Policy Checklist.

Statistics

For all statistical analyses, confirm that the following items are present in the figure legend, table legend, main text, or Methods section.

n/a	Confirmed	
☐	☒	The exact sample size (*n*) for each experimental group/condition, given as a discrete number and unit of measurement
☐	☒	A statement on whether measurements were taken from distinct samples or whether the same sample was measured repeatedly
☐	☒	The statistical test(s) used AND whether they are one- or two-sided<br>*Only common tests should be described solely by name; describe more complex techniques in the Methods section.*
☐	☒	A description of all covariates tested
☐	☒	A description of any assumptions or corrections, such as tests of normality and adjustment for multiple comparisons
☐	☒	A full description of the statistical parameters including central tendency (e.g. means) or other basic estimates (e.g. regression coefficient) AND variation (e.g. standard deviation) or associated estimates of uncertainty (e.g. confidence intervals)
☐	☒	For null hypothesis testing, the test statistic (e.g. *F*, *t*, *r*) with confidence intervals, effect sizes, degrees of freedom and *P* value noted<br>*Give P values as exact values whenever suitable.*
☐	☒	For Bayesian analysis, information on the choice of priors and Markov chain Monte Carlo settings
☒	☐	For hierarchical and complex designs, identification of the appropriate level for tests and full reporting of outcomes
☒	☐	Estimates of effect sizes (e.g. Cohen's *d*, Pearson's *r*), indicating how they were calculated

Our web collection on statistics for biologists contains articles on many of the points above.

Software and code

Policy information about availability of computer code

Data collection	The beta-values, which represent the proportion of methylated CpG, were estimated by the ratio of methylated/(methylated+unmethylated) signals and were obteined from patients with neurodegenerative diseases in the Gene Expression Omnibus database (GEO)
Data analysis	Raw data sets containing normalized methylation beta-values were laoaded into RStudio and filtered to include only regions of interest using the list of 18,293 pre-selected CpGs. Also with RStudio, we used a linear Bayesisn regression model, implmented through the "champ.DMP()" function from the "ChAMP" package, to define DMPs (differentially methylated positions) between cases and controls of each disease data set. The DDMPs were considere statistically significant if their adjusted p-values were less than or equal to 0.05; adjusted for multiple coparisons were obteined using the Benjamini-Hochberg method.

For manuscripts utilizing custom algorithms or software that are central to the research but not yet described in published literature, software must be made available to editors and reviewers. We strongly encourage code deposition in a community repository (e.g. GitHub). See the Nature Portfolio guidelines for submitting code & software for further information.

Data

Policy information about availability of data

All manuscripts must include a data availability statement. This statement should provide the following information, where applicable:

- Accession codes, unique identifiers, or web links for publicly available datasets
- A description of any restrictions on data availability
- For clinical datasets or third party data, please ensure that the statement adheres to our policy

All Methylation data used in this article were available in Gene Expression Omnibus (GEO) and were not generated in this study. Cases associated with neurodegenerative diseases used for comparison were based on the blood datasets available on GEO and included: Alzheimer's (GSE153712), Parkinson's (GSE111629), Multiple Sclerosis (GSE1006648), and Fragile-X Syndrome (GSE41273). Additionally, datasets with beta-values from brain tissue of patients with Alzhimer's (GSE72778), Parkinson's (GSE195834), and healthy controls were used for validation.

Research involving human participants, their data, or biological material

Policy information about studies with human participants or human data. See also policy information about sex, gender (identity/presentation), and sexual orientation and race, ethnicity and racism.

Reporting on sex and gender	The study adopted a exploratory approach, respecting the normalization and patient selection applied by the authors of each data set, without specific sub-division based on sex.
Reporting on race, ethnicity, or other socially relevant groupings	The study adopted a exploratory approach, respecting the normalization and patient selection applied by the authors of each data set, without specific sub-division in respect to race, ethnicity, or other socially relevant groupings.
Population characteristics	The study adopted a exploratory approach, respecting the normalization and patient selection applied by the authors of each data set, without specific sub-division based on ethnicity. In the first Alzheimer's cohort (GSE153712) 161 participants, aged over 60, included 91 females and 70 males. The second one (GSE72778) cohort had 65 participants (13 famele, 5 male, aged 58-114). In Parkinson's cohort (GSE111629, 335 participants (138 females, 197 males, aged 36-90) were included; the second Parkinson's cohort (GSE195834) 19 females and 21 males (ages 70-87) in a total of 38 patients. The Fragile X Syndrome cohort (GSE41273) had 9 participants (age 1-48, only males), while the Multiple Sclerosis cohort (GSE106648) comprised 140 participants (98 females, 42 males, aged 16-66).
Recruitment	This study was based on human DNA methylation data available in GEO.
Ethics oversight	This study was based on human DNA methylation data available in GEO.

Note that full information on the approval of the study protocol must also be provided in the manuscript.

Field-specific reporting

Please select the one below that is the best fit for your research. If you are not sure, read the appropriate sections before making your selection.

☒ Life sciences ☐ Behavioural & social sciences ☐ Ecological, evolutionary & environmental sciences

For a reference copy of the document with all sections, see nature.com/documents/nr-reporting-summary-flat.pdf

Life sciences study design

All studies must disclose on these points even when the disclosure is negative.

Sample size	We used the number of samples available in the datasets deposited on the GEO.
Data exclusions	Neurodegenerative diseases datasets that did not use Infinium HumanMethylation450K or Infinium Methylation EPIC to evaluate the methylation profile.
Replication	This study was based on data available on GEO.
Randomization	This study was based on data available on GEO.
Blinding	This study was based on data available on GEO.

Reporting for specific materials, systems and methods

We require information from authors about some types of materials, experimental systems and methods used in many studies. Here, indicate whether each material, system or method listed is relevant to your study. If you are not sure if a list item applies to your research, read the appropriate section before selecting a response.

Materials & experimental systems

n/a	Involved in the study
☒	☐ Antibodies
☒	☐ Eukaryotic cell lines
☒	☐ Palaeontology and archaeology
☒	☐ Animals and other organisms
☒	☐ Clinical data
☒	☐ Dual use research of concern
☒	☐ Plants

Methods

n/a	Involved in the study
☒	☐ ChIP-seq
☒	☐ Flow cytometry
☒	☐ MRI-based neuroimaging

Plants

Seed stocks	N/A
Novel plant genotypes	N/A
Authentication	N/A
